# Primary Healthcare Nurses’ Views on Digital Healthcare Communication and Continuity of Care: A Deductive and Inductive Content Analysis

**DOI:** 10.3390/nursrep12040091

**Published:** 2022-12-02

**Authors:** Ove Hellzén, Annika Kjällman Alm, Malin Holmström Rising

**Affiliations:** Department of Health Sciences, Mid Sweden University, 851 70 Sundsvall, Sweden

**Keywords:** digital communication, digital healthcare, interviews, nursing, person-centred care, qualitative methods

## Abstract

Primary healthcare in the Western world faces significant functional challenges, resulting in the implementation of digital communication tools. Nurses are key professionals in primary care and focusing on the impact of digital communication and continuity of care in primary care organisations is important. This qualitative descriptive study explores digital communication and continuity of care from primary healthcare nurses’ perspective. Data from individual semi-structured interviews with 12 nurses were collected; deductive and inductive content analyses were performed. Three descriptive categories emerged from the deductive (digital communication as interpersonal, information, and management continuities) and inductive (‘digital care does not suit everyone’, ‘new technology is contextually intertwined with daily work’, and ‘patient-positive aspects of digital information’) phases. Additionally, a structural risk of obscuration of patients’ needs by the contextual conditions emerged. To ensure digital communication-aligned continuity of care, compatible information technology systems should be developed. Allowing nurses to provide high-quality care based on their own values would enhance person-centred patient care.

## 1. Introduction

Healthcare in the Western world faces significant challenges, including reduced staffing, especially licensed nurses; fewer hospital beds; shorter hospital stays; and increased care intensity [1]. Moreover, demographic development and an aging population affects primary care due to declining resources and increasing hospital transfers. Therefore, patients with severe and chronic conditions are dependent on long-term contact with different care providers post-discharge because of the nature of their diseases; for example, patients with a diagnosis of heart failure [2].

Frequently, patients with chronic illnesses feel that they ‘fall between the cracks’ when care tasks are distributed to several care providers. Patients may experience a lack of information transfer and coordination between care organisations and staff [3]. In the event of deterioration, hospitalisation, or increased need for care in different environments, there is a risk of a lack of care continuity [4]. Continuity in patient care is a multifactorial concept that is influenced by environmental impact and communication, as well as patient, professional, and systemic factors [5]. Furthermore, it is described as the degree to which a series of care events are perceived as coherent, interconnected, and consistent with the patient’s medical needs and personal contexts [6].

Various care staff, from preventive to primary, secondary, and tertiary care, and in some cases, end-of-life and palliative care, tend to patients with long-term conditions throughout the care period [2]. The development of medical technology necessitates the formation of new organisations to ensure the fulfilment of patients’ needs. Therefore, enhanced knowledge on care within a system that is grappling with challenging transformations, such as an increasingly mobile staff, aging population, and long-term homecare and treatment for patients with severe chronic illnesses [7], is needed.

Literature on the concept of continuity of care within the context of primary care has been published, suggesting that this is an important concept in homecare as well [8]. Continuity of care is a core principle of primary care [9] and is an essential element of good primary care, along with coordination and patient-centred and integrated care [10]. The benefits include a better patient–provider relationship, increased patient satisfaction, improved uptake of preventive care, enhanced adherence to treatment, more accessible healthcare, and reduced healthcare use and costs [11,12]. 

Continuity of care is a core component in many international definitions of primary healthcare; however, it has been labelled as ‘hard to define’, and evidence of its benefits remain ambiguous [13]. Published reviews on the relationship between continuity of care and its outcomes have suggested that continuity of care is related to increased patient satisfaction [14].

The definition of continuity of care is dynamic owing to contextual factors such as the rising number of group practices and consumer movements. However, globally, a renewed interest in care continuity has been observed during the 21st century [15]. This is probably reflective of the ongoing healthcare organisational changes in Western countries, wherein the likelihood of patients consulting the same healthcare professional at repeated visits is reduced. For example, in Sweden, these changes include the introduction of a wider range of primary care providers, greater involvement of nurses, and the promotion of primary care specialist roles for both doctors and nurses [16]. 

Sweden, like many other countries, has a shortage of physicians and nurses, especially in primary care [17]. The demand for healthcare staff exceeds availability, leading to difficulties in recruiting skilled staff at health centres [18]. The interaction between care staff and patients can shift towards home-based care by adopting the developments in digital healthcare communication [19]. Digital healthcare communication is a prerequisite for municipalities and regions to offer cost-effective, qualitatively good, and equal care for citizens [20]. 

For patients, digital healthcare communication facilitates increased access to digitised information and health-related information via various websites, thereby transforming patient empowerment and patient-centred care [21]. This includes the ability to book appointments and receive reminders about renewing prescriptions [22]. Patients with chronic illnesses, including diabetes, heart disease, or cancer, are provided support via online forums and patient portals [23]. 

Primary care is considered to be at the ‘saturation point’ [24], facing increasing demand, reduced accessibility, and heightened patient dissatisfaction [25]. Consequently, provision of primary care through digitally focused methods is encouraged [26]. However, a digitalised primary healthcare system needs to focus on care continuity and person-centred care while emphasising the importance of flexibility among staff and healthcare units to adapt routines based on patients’ needs as a prerequisite for quality care [27]. Focusing on the impact of communication changes in primary care organisations on continuity of care is vital. 

### The Objective

Therefore, this study explores the concept of digital communication and the often-unclear concept of continuity of care from the perspective of primary healthcare nurses. 

## 2. Materials and Methods

### 2.1. Design

This study used an exploratory qualitative design [28]. Data were collected in accordance with Kvale and Brinkman’s guidelines [29] and deductive and inductive qualitative content analyses were performed. This study conformed to the Consolidated Criteria for Reporting Qualitative Research (COREQ) guidelines (in Supplementary Materials) [30]. 

### 2.2. Study Context

Primary care refers to medical treatment, nursing, preventive, and rehabilitative activities offered outside the purview of hospitals’ medical, technical, and special competence resources. Healthcare communication in primary care can be in physical and/or digital formats [31]. Currently, in Sweden, the shift in primary care approaches due to new challenges has led to nurses meeting patients, prioritising, consulting, communicating with patients and physicians, and making assessments with the increased support of digital technology, using digital communications tools such as video conferences, live chats and digital medical records to help patients get involved in their care and nursing.

### 2.3. Recruitment and Participants 

A purposive sampling strategy [28] was used to ensure heterogeneous samples regarding age, education, and length of work experience. The inclusion criterion for participation in the study was a minimum work experience of one year in primary care as a nurse. A secretary working in primary care distributed 20 invitation letters based on the inclusion criteria to nurses in the region. The invitation letters contained information about the study, an informed consent form and a reply form. Twelve nurses responded at once and were recruited. They were working at different health centres in the region. Each participant had more than two years of work experience in primary care as a district nurse (2–40 years; median 20 years). All participants had a master’s degree in district nursing, worked full-time, and were women. There was no prior relationship between the researchers and the participants.

### 2.4. Interviews 

**Figure 1:**
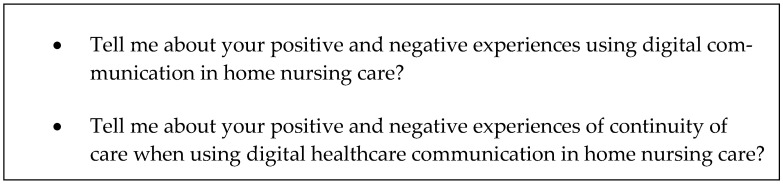
Interview guide.

Semi-structured interviews were conducted between September 2019 and January 2020, in line with Kvale and Brinkman’s guidelines [29]. An interview guide was used, and it was developed by the researchers. The interview guide was focusing on nurses’ experiences of using digital healthcare communication in home nursing care; Figure 1. During the interview, follow-up, and clarification questions such as ‘What did you feel/think/do in this situation?’, ‘Can you tell me more about that?’ and ‘Can you give an example?’ were asked. Most interviews were conducted in an undisturbed location at the participants’ workplaces, except one which was conducted at a participant’s home. The duration of the interviews was 20–40 min (median = 33 min). To ensure confidentiality, each participant was assigned a code comprising a capital letter and a number (A1, A2, etc.). The interview guide was piloted before the interviews. Then the interviews were conducted one by one, by the last author together with a PhD student, were audio-recorded, transcribed verbatim, and checked for accuracy. The transcribed material included a total of 150 pages of text. The interviewer was a female PhD, and all researchers were registered nurses with extensive experience of nursing and research.

### 2.5. Data Analysis

Qualitative content analysis was first performed according to Elo and Kyngäs’ description [32]; subsequently, Graneheim and Lundman’s description was applied [33]. Data analysis was performed in two distinct phases: deductive and inductive. This methodological integrative and iterative approach has been sparsely described in the nursing literature; however, Gjevjon et al. and Andersson et al. have described content analysis procedures that use theory as a grid for analysing text data [15,34]. Elo and Kyngäs opined that flexibility in terms of research design was an advantage of the content analysis method, and the use of deductive and/or inductive methods should be determined by the purpose of the research [32]. 

### 2.6. Deductive Phase

Deductive content analysis is useful when research on a phenomenon (e.g., continuity) benefits from further description, as in this study [32]. In the deductive phase, a structured categorisation matrix was constructed (Ibid). The matrix was based on Sparbel and Anderson’s concept of care continuity [5], and subsequently further expanded based on Haggerty et al.’s model [6] as three qualitatively different yet intertwined dimensions: interpersonal continuity, information continuity, and management continuity. The categorisation matrix was used as a lens during the analysis of the text and to form domains under which the data were sorted. Initially, the analysis began with repeated readings of the printouts to become familiar with and obtain an overview of the content. Subsequently, the printouts were carefully examined for content, and texts corresponding to the categorisation matrix were selected, coded, and transferred to the relevant matricial description categories. This phase of the analysis concluded with the first sub-goal of the study’s purpose; namely, to expand the nurses’ descriptions of their perceptions of continuity in care. 

### 2.7. Inductive Phase 

Inductive analysis [33] began with repeated open-minded readings of the transferred matricial text to gain an in-depth understanding that went beyond the earlier categorisation of the text in the deductive analysis. First, the data were repeatedly read to grasp the content and identify ‘units of meaning’ that corresponded to the study’s second sub-goals. Second, these ‘units of meaning’ were abridged to condense the meaning; however, the core remained intact. Third, these condensed ‘units of meaning’ were coded. The codes were abstracted, compared for differences and similarities, and sorted into subcategories and categories to gain further understanding and perspective on the nurses’ views on continuity of care relative to digital healthcare communication. These steps were performed for each identified domain in the deductive analysis; hence, three separate inductive analyses, one for each domain, were performed. Inductive analysis resulted in three identified generic categories. The analysis was led by first author (OH); the other authors (MHR and AKA) were the co-analysts. The codes, subcategories, and categories were deliberated and discussed by all authors throughout the analysis process, resulting in a consensus; see Table 1. 

### 2.8. Ethics

The participants received verbal and written information about the purpose of the study and gave written consent to participate. They were assured of confidentiality, with only the researchers connected to the project having access to the data, as per the guidelines of the Swedish Research Council [35]. The study conformed to the ethical guidelines of the Declaration of Helsinki (World Medical Association, 2013) [36] and was approved by the Swedish Ethical Review Authority (No: 2019-03353).

## 3. Results

Our deductive analysis revealed that the data reproduced the content of the three descriptive categories, which are summarised below in Table 2. Moreover, the analysis indicated that the relationship between the descriptive categories and the matrix’s dimensions of continuity of care was weak compared with that of the first deductive analysis. 

The results of the inductive analysis suggested that participants’ experiences and views of digital communication can be grouped into three categories: ‘digital care does not suit everyone’, ‘new technology is contextually intertwined with daily work’, and ‘patient-positive aspects of digital information’ (Table 3). Category one describes the perceived value linked to the patients. These values were associated with care relations and exemplified the facilitation of involvement in digital services. Categories two and three describe the organisation of care in terms of security and information quality, considered as fundamental based on the nurses’ involvement in digital communication. Collectively, these three themes comprehensively describe the ways in which the study participants experienced digital consultations and, consequently, digital primary care services.

### 3.1. Digital Care Does Not Suit Everyone

In this category, nurses mentioned that digital communication, like any other technology, encompasses both advantages and disadvantages. Their resistance to digital communication pertained to not only being unable to meet patients and colleagues face-to-face, it also included the fear of being alone and of losing contact with patients and colleagues. Although care meetings involved participation and the sharing of individual patients’ experiences, nurses were apprehensive that increased digital communication affected their ability to maintain care in accordance with their own values. A key advantage of digitalisation was that a digital meeting was considered a flexible and simple way to maintain contact with both patients and colleagues; thus, it could replace and/or supplement physical meetings. Participants emphasised that, regardless of the degree of digitalisation, the goal was to always provide the best possible care, irrespective of the location and mode of the meetings. 

Participants opined that digital interactions should complement physical interactions. Their descriptions clearly revealed that while increased digitalisation undoubtedly increased accessibility for both younger and older adults, it entailed a risk that the super-seniors may not be reachable, which was especially true for older women and for people with cognitive impairment or mental illness, and those whose mother tongue was not Swedish. Nevertheless, participants emphasised that, regardless of age and disability, patients usually knew more than might have been expected; many had smartphones or iPads at home, and many older adults are positive toward new technologies and preferred information via email instead of regular mail. 

Participants opined that in future primary care would require both physical and digital communication to maintain co-operation and dialogue with patients. Although digitalisation is inevitable in future patient care, identifying prospective patients for digital meetings and the requirements for a good interaction is necessary; hence, more information is needed. Digitalisation requires innovative thinking, and facilitating patients’ participation in their own care is crucial. Telecommunication systems enable multiparty calls with relatives for care planning as part of a coordinated individual patient care plan. For optimal communication, the possibility of video calls is required, as ‘you can see things that you do not hear’ (A12). Participants described the role of communication as ‘looking beyond the patient’(A6) and being present to confirm and strengthen the patient’s life situation. In cases where the patient could be observed, being ‘extra sensitive to what the person is saying so that you catch symptoms’(A4) required nurses to develop a ‘clinical hearing’(A12).

### 3.2. New Technology Is Contextually Intertwined with Daily Work

This category represents participants’ descriptions of digital technology as vulnerable yet having a positive effect on daily work. The interviews highlighted several factors which formed the basis for participants’ positive experiences of digitalisation in association with direct care practice. In addition to accessibility, digital communication refers to fast and simplified communication channels in collaboration with doctors, which was an important aspect of increased patient safety and workplace safety factors. Although many patients felt that they needed to consult a doctor even though consultation with a nurse was sufficient, doctor consultations were necessary, especially for chronically ill patients who needed physical appointments. Therefore, the assessment of the patient’s condition was based on a national standardised form of triage which helps to ‘filter out patients’(A4) to facilitate adequate doctor consultations. The ability to distinguish symptoms through telephonic interaction makes available medical and nursing resources, especially for older patients and those who need more time. 

Participants emphasised that digital communication should never replace human interactions, and that technology should complement traditional care by creating multiple communication channels and uncluttering resources. Furthermore, participants stressed that digitalisation offered rapid feedback, which provided them a sense of participation in care and work security. Although participants viewed digitalisation positively, they highlighted the importance of physical interactions: ‘it’s easier to see and read body language about how a person feels at a physical encounter’(A6). Participants had positive experiences of assessments via digital communication; however, they felt that it was inadequate since they preferred to physically examine the patients. Moreover, much time was spent contacting the appropriate person for information access. Sometimes, it took weeks to obtain the requested information; this was perceived as frustrating. Participants felt that increased digitalisation increased their administration work and gave them the impression that they must be constantly available; this was perceived as a stress factor that led to more routines to handle the situation. 

Patients with complex care needs and multiple diagnoses requiring home care service were often the basis for participants seeking collaboration with different care professions. The contact networks and processes on which they depended on for work communication and co-operation required planning and coordination of care interventions. Digital coordination and planning facilitated efficient work and saved time by reducing to-and-fro conveyance between different health centres, which enabled more sustainable home healthcare. Furthermore, digital communication is economically advantageous for patients since they do not have to drive their own car or use a transport service ‘which is not free either’(A10). Some participants considered that it would be desirable to conduct follow-ups of the health interviews digitally. During the interviews, they emphasised that they preferred quick feedback after the completion of the assessment, in order that the patient did not have to wait for information. Moreover, participants felt that digital communication was easier than a long commute for a half-hour visit for older and less mobile patients. 

Participants expressed concerns about the difficulties that may arise due to technical problems in the new technology that could lead to information loss. Several staff members lacked digital skills and became confused in case of problems, and digital tools are rendered more vulnerable in such situations. Lack of education and technical support was perceived as a weakness. Concerns were expressed on the maintenance of data integrity and confidentiality, especially for both care responsibility and personal responsibility; care responsibility refers to ensuring a safe meeting, and personal responsibility refers to the agreements reached during the interaction.

### 3.3. Patient-Positive Aspects of Digital Information 

The patient-positive aspects of digital information are associated with security issues and information quality. Participants considered that digitalisation improved care both for themselves as professionals and for the patients. The availability of improved patient information led to saved time and streamlined care, which benefited both care providers and care recipients. Participants opined that simplification of documentation and assessment contributed to improved patient safety. The improvement of referral management for specialist care has been significant; for example, photographs of wounds and referral documents are attached to digital patient records, leading to faster doctor assessments and prescriptions. 

A disadvantage of the increasing digitalisation in society was that the participants were questioned more often by well-informed patients and/or relatives who had searched online for information on symptoms, diseases, etc. before visiting the healthcare centre. To avoid this, participants emphasised the importance of using the e-health platform 1177, which is recommended for patients and relatives. This platform contributes to increased patient safety as it reduces the risk of spreading incorrect information to patients. Participants acknowledged that digitalisation necessitated a rapid change in their work with the introduction of new routines and working methods. Thus, digitalisation had increased their workload because computer systems were not always integrated and compatible with each other. Furthermore, participants stressed the disadvantages of different journal systems, which forced them to maintain certain notes in folders.

Participants highlighted the significant advantages of digitised patient records: that they were easy and clear to read, and that all necessary patient information could be accessed on their work computer. Owing to faster and seamless data entries, participants could access the details entered by the other healthcare providers. Digital medical records were considered to be more efficient and secure, because they are more easily accessed by the patient; moreover, all relevant patient information is collected to minimise the risk of misjudgements. These facts were emphasised as an advantage since nurses needed to be careful about their entries in the medical records. Additionally, participants highlighted the advantage of standardised assessment during conversations, indicating that all internal medical record systems and intranets in computers, telephones, radiology departments, and counselling centres should have the same assessment criteria as the e-health platform 1177.

## 4. Discussion

This study aimed to describe primary care nurses’ perceptions of digital communication and continuity in care. The results of the content analysis revealed that the data mainly reflected participants’ comprehension of continuity of care. However, the relationship between the outcomes was weak; that is, the participants’ views on the relationship between continuity of care and digital communication could be understood based on three categories: the importance of personal care, new technology affecting daily work, and patient-positive aspects of digital information. This finding was interpreted as an alternative and sometimes contrasting perspective to Sparbel and Anderson’s view of the continuity of care [5], which in conjunction with the identified categories from the inductive analysis provided an increased understanding of the care provided.

### 4.1. ‘Interpersonal Continuity’ vs. ‘Personal Care Does Not Apply to Everyone’ 

The deductive analysis reveals the impact of the descriptive category ‘Interpersonal continuity’ on the nurses’ perceptions of the relationship’s significance on their methods of reaching the person behind the label ‘patient’ for providing person-centred care. Conversely, the findings of the category ‘Digital care does not suit everyone’ suggest that descriptions of digital communication-based care were not adaptable for everyone; thus, person-centred care could not always be offered. Participants’ descriptions can be described as two conflicting dualities: (1) the ability and willingness to work in accordance with a person-centred approach and (2) descriptions of the impact of structural and contextual conditions that made it challenging to offer person-centred care and risked excluding specific patient groups. ‘Interpersonal continuity’ is the dimension of continuity in care which is most appreciated by both staff and patients [37]. Women, older adults, and patients with long-term illnesses emphasise the importance of ‘interpersonal continuity’ [38]. Continuity in care also is reportedly associated with improved patient outcomes [27] and increased patient satisfaction [39]. However, the value of ‘interpersonal continuity’ has decreased over the last decade because of the increased use of technology in primary care [40]. 

The introduction of digital alternatives to complement to face-to-face interactions illuminates the importance of understanding the impact of technology, such as the possibility of older adults having access to their healthcare needs. Digitalisation of healthcare has changed both the practice and experiences of care relationships and has led to fewer physical interactions between patients and care staff [41]. In nursing theory, a caring patient-nurse relationship is a fundamental value [42]. Patient-nurse relationships are considered central to patients’ health, well-being, and involvement in care [43]. Therefore, from the patient’s perspective, it is important that nurses are present both physically and emotionally during patient interactions [44]. Fagerberg reported that reduced face-to-face interactions affected nurses’ ability to provide high-quality patient care [45].

Our analysis indicated that participants believed digital communication to be a source of opportunities in primary care; however, questions about quality, access, and equality in primary care with respect to the older adults and chronically ill patients remained. The participants emphasised that the importance of relationships was significant in digital care, and that involvement in technological development was facilitated by the existence and maintenance of ongoing patient relationships. Accordingly, close and personal caring relationships may be a prerequisite for providing good, accessible, and equitable digitalised healthcare services.

### 4.2. ‘Management Continuity’ vs. ‘New Technology Is Contextually Intertwined with Daily Work’

Other studies have reported similar results. Wolf et al. deduced that care environments that strongly focus on routines and lack the ability for patient dialogue affect nurses’ abilities to fulfil their moral convictions and purpose of nursing [46]. Our study revealed that participants were conflicted between ideals (how care should be [the theory]) and reality (the structures of the care environment and the daily activities). Home care patients with complex care needs, such as older people or persons with mental health problems, often constitute the basis for increased collaboration with different care professions. Management continuity ensures that care conditions are aligned to patient needs. Studies have reported that patients value easy and quick access to support [47], flexible and responsive care [48], and the possibility of care planning and coordinated transitions [49]. The contact networks and processes require planning and coordination for care interventions. Our results indicated that contextual conditions challenge the participants’ ability to maintain care in accordance with their beliefs of good care practices. This may be problematic; Bridges et al. have reported that nurses who were unable to provide good quality care to patients were more likely to report experiences of moral distress, guilt, and frustration, and that these experiences might result in nurses distancing themselves from the patient to protect themselves [50]. Our results indicated a need for greater awareness of the impact of contextual conditions on nursing care. Therefore, furthering knowledge on the optimal structuring care context is important for providing contextual improvements.

### 4.3. ‘Information Continuity’ vs. ‘Patient-Positive Aspects of Digital Information’

Our results indicated that digital communication improved care for both participants and patients. Moreover, the content and access to patient information have improved. The descriptive category ‘information continuity’ reflects nurses’ perceptions of the patient-positive aspects of digital information, aspects related to security issues, quality of information, and its significance for the performance of care. Participants stated that digitalisation rapidly transformed their work; the introduction of new routines and working methods led to increased personal responsibility, and they worked faster; they sometimes felt that they could not or did not care about things that they might have forgotten.

The complexity of primary care requires it to be based on clinical assessments wherein patients’ experiences and preferences are considered. The current contextual conditions appeared to limit the participants’ ability to establish interventions that deviated from patients’ needs. In the descriptive category ‘patient-positive aspects of digital information’, the participants described their care efforts as those based on the individual’s needs and preferences and designed to strengthen the individual patient’s health process. Conversely, information dissemination and counselling were aspects reflected in participants’ descriptions of care; these appeared as contextual relationships that are unsupported by other types of nursing interventions. This was mostly described as a lack of face-to-face time or flexible follow-up systems. Consequently, the descriptions suggested a limitation in nurses’ ability to include patients’ preferences in the development or delivery of care interventions.

### 4.4. Methodological Considerations 

According to Guba, the credibility of this type of study is best evaluated within its own framework, thereby strengthening the possibility of credible results and interpretations [51]. This requires exploring the complexity of the phenomena under study (Ibid) and discussing the weaknesses and strengths of the study. The present study was based on semi-structured interviews conducted with 12 nurses from different regional health centres, and the interviews were rich in information since participants elucidated their experiences of using digital communication in healthcare. The analytical process was iteratively performed in two phases (deductive and inductive); it was not a repetitive mechanical task, rather it was a reflexive process with the goal of being conscientious to the collected empirical data. The results of this study cannot be generalised because the intention of qualitative research is not generalisation. Rather, these findings can be recontextualised and transferred to similar healthcare settings outside of Sweden. To enable the reader to assess the study’s credibility and reliability as well as its relevance to similar environments, adequate quotations are presented in Table 1 and Table 2. 

## 5. Conclusions

Our results indicate that primary care nurses were conflicted between ideals (how care should be [the theory]) and reality (the structures of the care environment and the daily activities). Digital communication improved care for both participants and patients. Moreover, the content and access to patient information have improved. However, the contextual conditions wherein the concepts of care continuity and digital communication pose challenges to primary care nurses’ potential for providing quality care in accordance with their own values. A structural risk that patients’ needs and preferences might be obscured by prevailing contextual conditions was observed; therefore, further research is required. Various compatible IT systems should be developed for ensuring care continuity in a systematic manner. This may provide nurses with increased opportunities for person-centred care, wherein patients’ experiences are considered in addition to their own care values. To ensure digital communication-aligned continuity of care, compatible information technology systems should be developed. Allowing nurses to provide high-quality care based on their own values would enhance person-centred patient care. 

## Figures and Tables

**Table 1:** Example of analysis process in inductive phase.

Meaning Unit	Code	Subcategory	Category
‘When it comes to digital care encounters, I think there is a fundamental scepticism, just like I had before, that it is not genuine and it is not genuine, does not suit everyone’. (A6)	Not genuine encounters	Scepticism towards digital meetings	Digital care does not suit everyone
‘Digital care is good, it works well, we don’t have to travel long distances, we see each other anyway, we can ask questions and have a dialogue, everything works, I use it as often as I can. I can chat with doctors, easily access medical records and read if I need to… you can even record sounds that is, heartbeats, breathing sounds and… it’s great. It’s so flexible’. (A1)	Decreased need to travel	Online care meetings with patients, close relatives, and healthcare	New technology is contextually intertwined with the daily work

**Table 2:** Summary of the deductive analysis process departing from the matrix’s dimensions.

	Descriptive Categories	
Digital Communication as Interpersonal Continuity	Digital Communication as Information Continuity	Digital Communication as Management Continuity
‘…it is certainly possible to teach them, they are not completely… they, almost everyone, they have mobile phones now. There are actually a lot of the older ones who also ask “can you not send that by email instead” so that it is not just younger, but I think there are quite a few 80+ are also properly digitized’. (A7)	‘Yes, so it’s also digital, so I think it works well, you do not have to go all the trips between, we see each other anyway, we can ask questions and then we have no sound, while we listen to what they say, and so they want ask a question, then you start the sound, then everything works, then it’s smooth’. (A3)	‘… something that is appreciated in any case… I think… it is the Facebook posts, where we advertise for vaccination that now it is in progress and what the Christmas closure looks like and which, which groups are in progress and swimming course and osteoarthritis school and them the pieces… I think it is appreciated and it… I think is a good way to go get information out’. (A4)

**Table 3:** Summary of the inductive analysis process departing from the matrix’s dimensions.

	Descriptive Categories	
Digital Care Does Not Suit Everyone	New Technology Is Contextually Intertwined with the Daily Work	Patient-Positive Aspects of Digital Information
‘The negative is the lack of the human… I mean, depending on the case, how should I as a patient feel if I live out in sparsely populated areas, I would not feel confident that… that I got the help I need if I could just talk to someone via video…. no way!’ (A8) ‘In the event of a major cognitive impairment, there can be problems with a digital meeting, but at meetings for a coordinated individual patient plan, you always have someone on site at home. It gets easier when someone is sitting there’ (A9) ‘I have to think about how I express myself and how it is received when I do not see the facial expression on the person’ (A12)	‘We need to get all the information about the patient to be able to make a good health assessment as the whole person is important. It is for the patient’s sake that we need all the information and to streamline care so we do not have to call around for information’. (A5) ‘…it can be wrong if you write suspicions about cancer diagnosis and then it is not communicated with the patients but they read about it themselves. I think a lot about how I write now that they can read for themselves. I make sure to be clear and nicely written when they can read themselves’ (A11) ‘The paper records were only in one copy, so if it disappeared, no one had access to it. Now that problem does not exist at all. Different actors have access to it at the same time. From the beginning, I thought it was hard, but now I have a good basis when I go in and check each test answer separately and see immediately if something deviates.’ (A10)	‘Contact with the doctor usually takes place via chat. It facilitates assessment, follow-up and medication change. He (the doctor) changes it, I think it was very flexible’ (A1) ‘Even if the information is necessary, it should be presented in a form so that we can adapt to it. If not, if the overload of information continues, it will have negative consequences for the quality of care’. (A6) ‘A whole new world opened up when we switched from phone to video meeting, when I could see the person and have a real conversation. It is easy to hand over the floor and to lead the meeting when I see the people who participate, if they understand or want to say something more’ (A2)

## Data Availability

The data presented in this study are available on request from the corresponding author.

## Supplementary Materials

The following supporting information can be downloaded at: https://www.mdpi.com/article/10.3390/nursrep12040091/s1, COREQ (COnsolidated criteria for REporting Qualitative research) Checklist.
